# Recent Progress in Surgery

**Published:** 1865-03

**Authors:** 


RECENT PROGRESS IN SURGERY. The Annual Address delivered before the Massachusetts Medical Society, May 25th, 1864. By JOHN MASON WARREN, M.D. Boston: 1864.

This is a well-written and instructive address, printed on excellent type and paper. It gives a clear exposition of the progress made in the pathology and practice of Surgery during the last quarter of a century.

